# Automatic Classification of GI Organs in Wireless Capsule Endoscopy Using a No-Code Platform-Based Deep Learning Model

**DOI:** 10.3390/diagnostics13081389

**Published:** 2023-04-11

**Authors:** Joowon Chung, Dong Jun Oh, Junseok Park, Su Hwan Kim, Yun Jeong Lim

**Affiliations:** 1 Department of Internal Medicine, Nowon Eulji Medical Center, Eulji University School of Medicine, Seoul 01830, Republic of Korea; 2 Department of Internal Medicine, Dongguk University Ilsan Hospital, Dongguk University College of Medicine, Goyang 10326, Republic of Korea; 3 Department of Internal Medicine, Digestive Disease Center, Institute for Digestive Research, Soonchunhyang University College of Medicine, Seoul 04401, Republic of Korea; 4 Department of Internal Medicine, Seoul Metropolitan Government Seoul National University Boramae Medical Center, Seoul 07061, Republic of Korea

**Keywords:** capsule endoscopy, artificial intelligence, automatic organ classification, automated machine learning

## Abstract

The first step in reading a capsule endoscopy (CE) is determining the gastrointestinal (GI) organ. Because CE produces too many inappropriate and repetitive images, automatic organ classification cannot be directly applied to CE videos. In this study, we developed a deep learning algorithm to classify GI organs (the esophagus, stomach, small bowel, and colon) using a no-code platform, applied it to CE videos, and proposed a novel method to visualize the transitional area of each GI organ. We used training data (37,307 images from 24 CE videos) and test data (39,781 images from 30 CE videos) for model development. This model was validated using 100 CE videos that included “normal”, “blood”, “inflamed”, “vascular”, and “polypoid” lesions. Our model achieved an overall accuracy of 0.98, precision of 0.89, recall of 0.97, and F1 score of 0.92. When we validated this model relative to the 100 CE videos, it produced average accuracies for the esophagus, stomach, small bowel, and colon of 0.98, 0.96, 0.87, and 0.87, respectively. Increasing the AI score’s cut-off improved most performance metrics in each organ (*p* < 0.05). To locate a transitional area, we visualized the predicted results over time, and setting the cut-off of the AI score to 99.9% resulted in a better intuitive presentation than the baseline. In conclusion, the GI organ classification AI model demonstrated high accuracy on CE videos. The transitional area could be more easily located by adjusting the cut-off of the AI score and visualization of its result over time.

## 1. Introduction

Capsule endoscopy (CE) is an innovative diagnostic method that enables the exploration of the small bowel, which cannot be accessed by esophagogastroduodenoscopy or colonoscopy. However, it takes substantial efforts to find only a few lesions out of hundreds of thousands of images recorded over 4 to 10 h in the small bowel. Therefore, a computer-assisted diagnostic approach is helpful for CE readings, and many studies have reported positive results in detecting small bowel diseases [1,2,3,4,5,6,7,8,9,10,11,12,13]. The small bowel is filled with feces, bile, and bubbles even after bowel preparation; therefore, many unsuitable images for diagnoses are captured [14]. Prior studies deliberately chose accurately taken mucosa over low-quality images, such as blurry and dirty images and images with partially visible pathological lesions. As a result, deep learning models were developed using only 14–930 images per 1 CE case, which could not represent the entire video [6,7,8,9,10,11,12,13]. Since these models show disappointing outcomes in actual CE videos, which are filled with inadequate images, they have not yet been applied in clinical practice [15,16]. Therefore, an AI model that can be used across entire CE videos, regardless of the image’s quality, is constantly needed to reduce clinicians’ reading time.

Identifying each GI organ from hundreds of thousands of images is the first step in CE reading, which can take several minutes in some circumstances [17,18]. Automatic GI organ classification can speed up reading, aid in locating pathologic lesions, and calculate the bowel transit time automatically.

In this study, we developed a deep-learning model for classifying GI organs using a no-code platform. A no-code platform, an automated machine learning tool, was a specialized tool for automatic model selection, quickly identifying the best hyperparameters, and achieving excellent classification performance without computational knowledge [19,20,21]. Clinicians can readily create deep learning models using this, and their performance was comparable to that of conventional models [20,21,22]. To represent the full-length video, we extracted images evenly throughout the video and added a preprocessing step to remove inappropriate images, which comprise a substantial portion of the actual CE video. Finally, we improved diagnostic performance and enabled intuitive judgments of the transitional area by adjusting the prediction probability cut-off and visualizing the result.

## 2. Method

### 2.1. Data Collection and Labeling

We used a dataset consisting of 154 CE videos collected retrospectively from 2 university hospitals (Dongguk University and Soonchunhyang University) with the approval of the individual, institutional review board. CE was performed between 2016 and 2021 using PillCam SB3 (Medtronic, Minneapolis, MN, USA).

**Figure 1:**
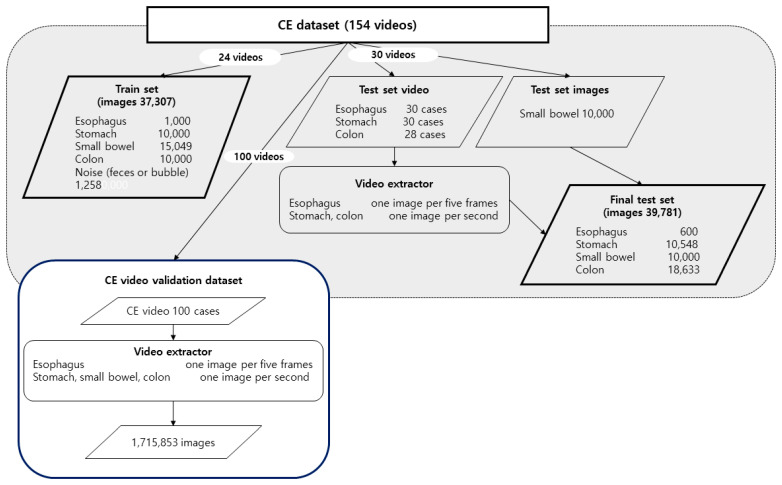
Data collection flowchart.

After data anonymization, fifty-four normal CE cases were classified into the esophagus, stomach, small bowel, and colon by three experts (LYJ, ODJ, and CJW) with over five years of experience in gastroenterology using a dedicated viewer (Rapid Reader ver. 8.3, Medtronic, Minneapolis, MN, USA). The majority consensus of the three experts defined the ground truth. In total, 1258 noise images with bubbles and feces were selected to develop the pre-procession algorithm. Then, the fifty-four labeled videos were randomly divided into the training dataset (24 cases) and the test dataset (30 cases) (Figure 1).

We randomly chose 10,000 esophageal images, 15,049 small bowel images, 10,000 colon images, and 1258 noise images from 24 CE videos in the training dataset. The 30 CE videos of the test dataset consisted of 30, 30, 30, and 28 videos for the esophagus, stomach, small bowel, and colon, respectively. We arbitrarily extracted images from the video using the following method to reduce biases caused by the expert’s manual selection and minimize the loss of video-recorded information. Each video extracted one image per five frames of the esophagus and one image per second of the stomach, small bowel, and colon. Because the small bowel video contains the most extensive and heavily repeated images, 10,000 images were randomly selected for practical model testing. As a result, the test dataset included 600 esophageal, 10,548 stomach, 10,000 small bowel, and 18,633 colon images.

Before applying the AI model to CE videos, 3 gastroenterologists classified 4 GI organs and labeled lesions (“normal”, “bleeding”, “inflamed”, “vascular”, and “polypoid” lesion) [22] on 100 full-length CE videos using a dedicated viewer. Then, the above-described video extraction technique was used to extract images. The data collection process is illustrated in Figure 1.

### 2.2. Automated Deep Learning Tool for Model Establishment

A no-code deep learning tool was used in this study. Neuro-T version 2.3.3 (Neurocle Inc., Seoul, Republic of Korea) automatically builds effective deep convolutional neural networks for image recognition and classification using a software algorithm that analyzes the features of the dataset and self-discovers optimal hyperparameters, thus making it easy for non-experts to build the best models [19,20,21,22].

The hardware system for establishing the Neuro-T-based model included four GeForce RTX 2080 Ti GPUs, dual Intel^®^Xeon CPUs, and 256 GB RAM.

### 2.3. Data Preprocessing

The no-code tool provides user-friendly preprocessing and training options [19,20,21,22]. This provides image resizing conversion for input images and allows users to select multiple modes for the resizing transformation of the input data. In this study, all images were resized to a resolution of 512 × 512 pixels before training. Neuro-T also offers options for selecting the level of training time based on the available graphic processing units (with four categories: fast and levels 1, 2, and 3) and a range of inference speeds based on batch size (three categories: levels 1, 2, and 3). We conducted several experiments to determine the best-performing deep learning model based on various convolutional neural network structures.

### 2.4. Deep Learning Model Development

Since randomly extracted images may include inappropriate images containing feces or bubbles, we added a noise control process to filter them. Then, the GI organ classification algorithm was developed to divide images into four GI organs: the esophagus, stomach, small bowel, and colon.

**Figure 2:**
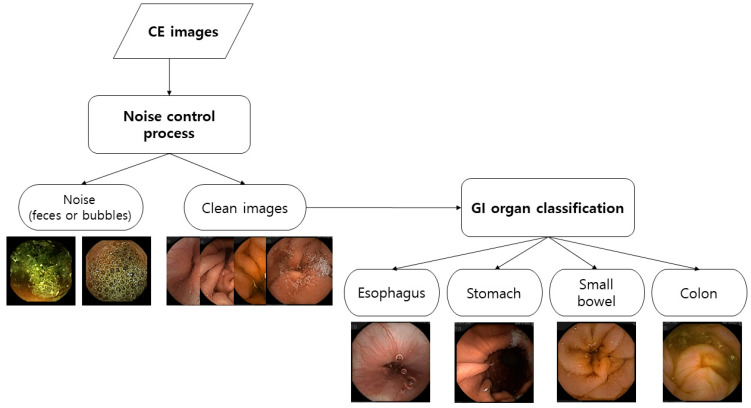
Model development flowchart.

For the noise control algorithm, 1263 images labeled as noise (feces and bubbles) and 36,044 images of the training dataset without any comment of noise were uploaded to Neuro-T. After selecting data preprocessing options with resizing and normalization, the models were trained in their specified self-learning manner (batch size of 20, 16 epochs, 6 layers, and a global learning rate of 0.00146). Then, the images filtered from the first algorithm were used to develop the GI organ classification algorithm using Neuro-T in the same way (batch size of 36, 33 epochs, 6 layers, and a global learning rate of 0.00146). After model development, it was applied to the test dataset and finally validated for the 100 CE videos. Figure 2 illustrates the model development process.

### 2.5. Statistics, Primary and Secondary Objectives

The primary objective was to evaluate the accuracy of this model applied to actual CE videos. Additional performance metrics were precision, recall, specificity, negative predictive value (NPV), F1 score, and ROC curve. The prediction probability was defined as the AI score in this study. The secondary objective was to identify the transitional area between the GI organs. We presented the transitional area via the condensed results by adjusting the AI score and visualization over time. A paired T-test and independent T-test were conducted to compare performances before and after adjusting the AI score cut-off and to compare the accuracy of each small bowel disease, respectively. *p*-values of <0.05 were considered significant, and all statistical tests were 2-sided. The analysis was performed using SPSS ver. 18.0 for Windows (SPSS Inc., Chicago, IL, USA).

## 3. Result

### 3.1. Diagnostic Performance of the GI Organ Classification AI Model Using the No-Code Tool-Based Deep Learning Algorithm

**Figure 3:**
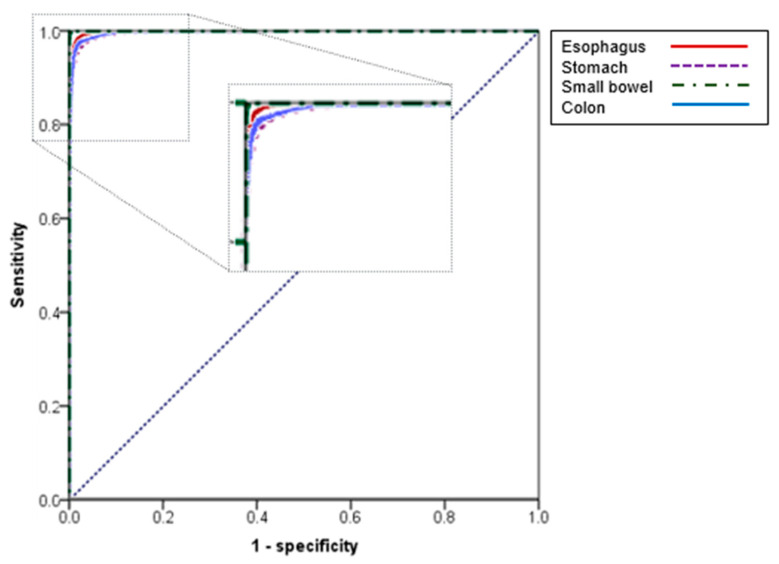
Receiver operating characteristic curve of the convolutional neural network for classifying GI organs.

**Figure 4:**
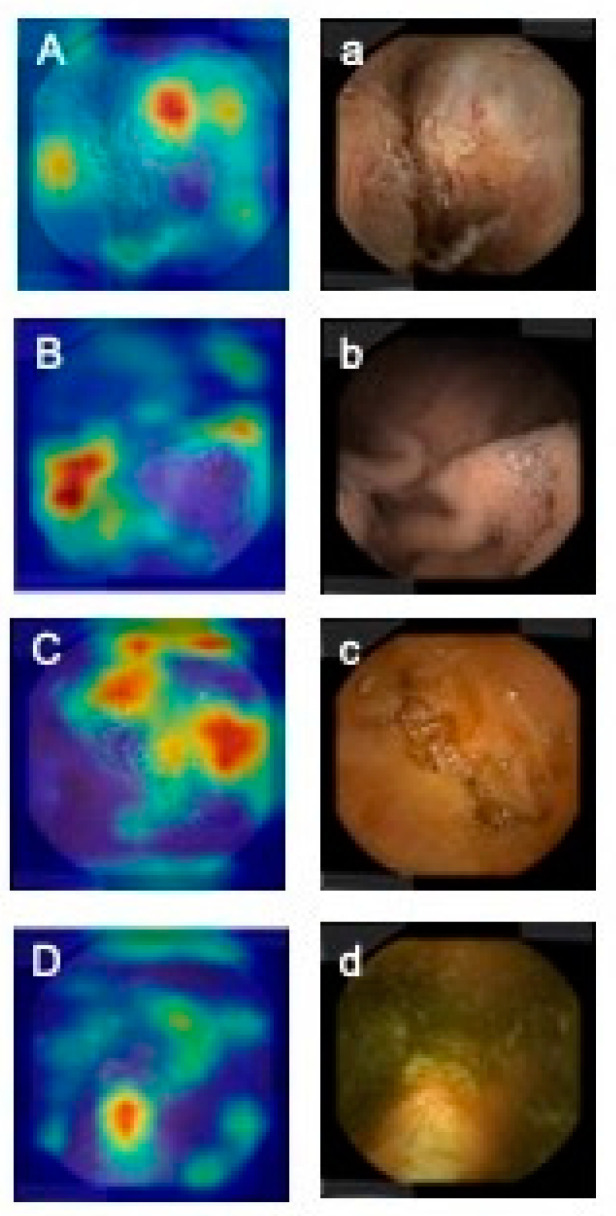
Representative cases of correctly predicted classes in the test dataset using the no-code tool. (**Left**) Gradient-weighted class activation mapping image. (**Right**) White-light imaging capsule endoscopic image. (**A**,**a**) esophagus; (**B**,**b**) stomach; (**C**,**c**) small bowel; (**D**,**d**) colon.

**Figure 5:**
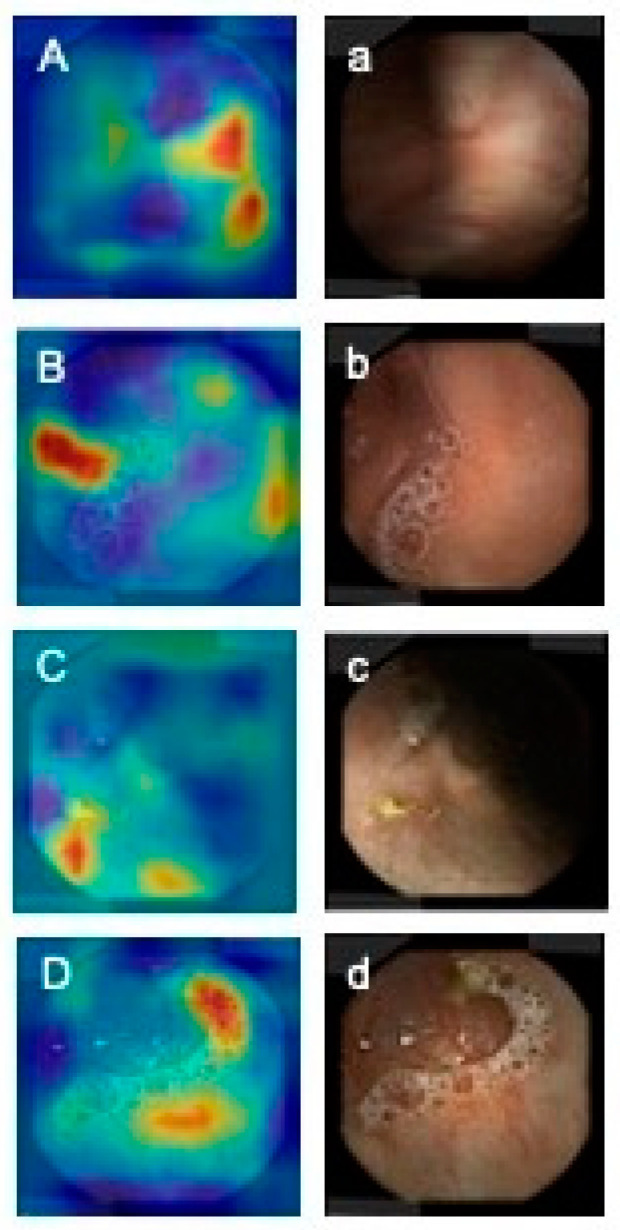
Representative cases of incorrectly predicted classes in the test dataset using the no-code tool. (**Left**) Gradient-weighted class activation mapping image. (**Right**) White-light imaging capsule endoscopic image. (**A**,**a**) Esophagus, but predicted as the stomach; (**B**,**b**,**C**,**c**) stomach, but predicted as the small bowel; (**D**,**d**) colon, but predicted as the esophagus.

A total of 35,535 clean images proceeded to the GI organ classification AI model after the noise control process eliminated 4246 of 39,781 images in the test dataset. This model demonstrated an overall accuracy of 0.98, recall of 0.97, specificity of 0.99, and precision of 0.89 for classifying GI organs, and the overall AUROC was 0.941 (Table 1). The AUROC for each GI organ is shown in Figure 3. The accuracy and an F1 score for each organ ranged from 0.97 to 0.99 and 0.78 to 0.98, respectively. The small bowel showed excellent accuracy (0.99) and an F1 score (0.98). The esophagus had the lowest F1 score (0.78) due to its low precision (0.66). The mucosa was highlighted in both accurately and inaccurately predicted images (Figure 4 and Figure 5).

### 3.2. CE Video Application of the GI Organ Classification AI Model

The 100 CE videos produced 1,715,852 images by video extraction (5400 in the esophagus, 229,618 in the stomach, 1,206,033 in the small bowel, and 274,801 in the colon). During the noise control process, 311,525 (18.2%) images were eliminated (60 in the esophagus, 11,644 in the stomach, 218,420 in the small bowel, and 81,525 in the colon). The number of images used in the video analysis is listed in Supplementary Table S1.

The GI organ classification AI model was applied to the 1,404,203 images and showed an overall accuracy of 0.92, recall of 0.89, specificity of 0.96, precision of 0.67, and NPV of 0.90 for identifying GI organs (Table 2). The esophagus and stomach showed a high accuracy of 0.98 and 0.96, respectively, but the precision of the esophagus and colon were poor at 0.26 and 0.54, resulting in a low F1 score of 0.33 and 0.64, respectively. The small bowel showed an accuracy of 0.87, specificity of 0.98, precision of 0.98, and F1 score of 0.89.

When each image’s AI score (i.e., predictive probability) was limited to 99.9% or more, approximately 43.7% of entire images were eliminated. The outcomes for the remaining 904,906 images revealed that every performance metric in every GI organ increased: 0.96 accuracy, 0.94 recall, 0.98 specificity, 0.85 precision, and 0.95 NPV in total (Table 2). Supplementary Table S2 shows that the 99.9% cut-off application significantly improved most performances in each GI organ.

### 3.3. Performance of the GI Organ Classification AI Model by Diseases in CE Videos

The 100 video cases included common small bowel diseases such as bleeding, inflammation, abnormal vascularity, and polyp, and our model’s performance did not differ significantly depending on conditions other than blood (Table 3 and Supplementary Table S3). The cases with “blood” had lower accuracy than other diseases, but increasing the AI score threshold to 99.9% complemented its performance.

### 3.4. Visualization of the GI Organ Classification and the Transitional Area

**Figure 6:**
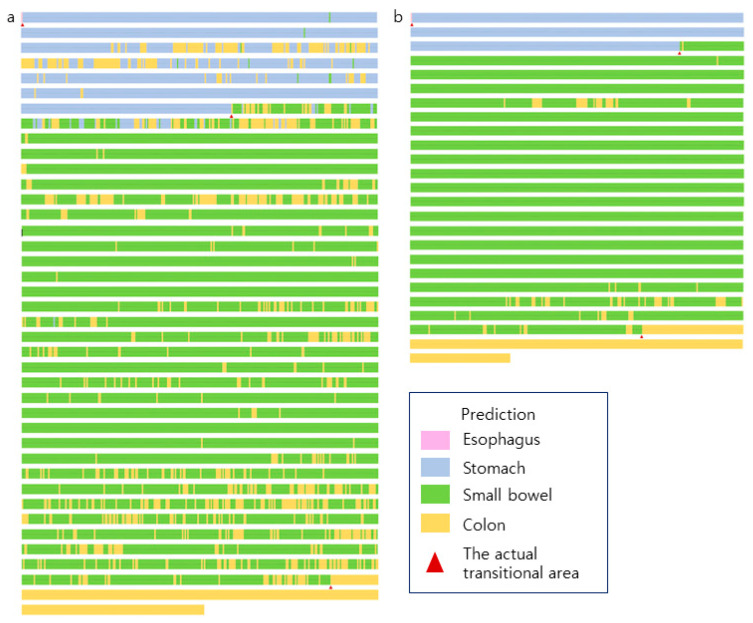
Graph visualizing the results of GI organ classification AI model over time. (**a**) Graph of entire images taken from a normal CE video. (**b**) Graph of condensed images by setting the AI score’s cut-off to 99.9%.

**Figure 7:**
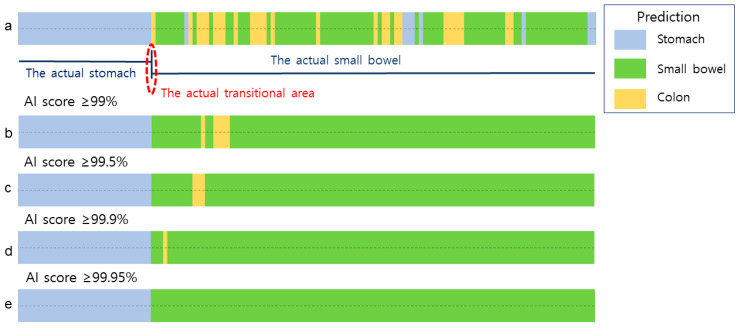
The transitional area between the stomach and small bowel. (**a**) A graph visualized by extracting the actual stomach small bowel transitional area. After the actual transitional area, the parts predicted to be the small bowel and the colon are alternatively observed. (**b**) Cut-off of AI score ≥ 99%; (**c**) AI score ≥ 99.5%; (**d**) AI score ≥ 99.9%; (**e**) AI score ≥ 99.95%. Setting a cut-off clarifies the transitional area.

**Figure 8:**
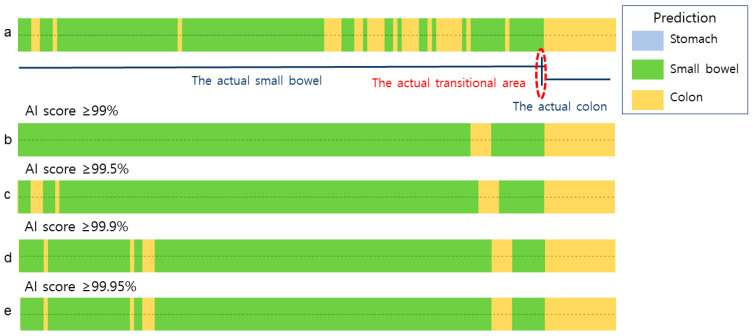
The transitional area between the small bowel and colon. (**a**) The graph was visualized by extracting the actual small bowel colon transitional area. Before the actual transitional area, the parts predicted to be the small bowel and the colon are alternatively observed. (**b**) Cut-off of AI score ≥ 99%; (**c**) AI score ≥ 99.5%; (**d**) AI score ≥ 99.9%; (**e**) AI score ≥ 99.95%. Setting a cut-off clarifies the transitional area.

We visualized the prediction results by listing them in a time sequence. Despite our AI model’s high performance, there is a limit to detecting the transitional area at once when applied to all images of each CE video because the result is not sequential but rather scattered (Figure 6a). Therefore, we attempted to present the transitional area by compressing the images by gradually increasing the cut-off of the AI score. The transitional area was visualized more clearly without a loss of information as the threshold increased to 99.9% and 99.95% from the default (Figure 7 and Figure 8). Figure 6 compares the clarity of the GI organ classification before and after the AI score control.

## 4. Discussion

In this study, we developed an AI algorithm that automatically classifies GI organs from images taken by capsule endoscopy, and it demonstrated good performance. Attempts to distinguish GI organs from capsule endoscopy images have been steadily made. Lee et al. focused on the fact that each digestive organ shows different patterns of intestinal contraction and used it for color change feature analyses based on HSI (hue, saturation, and intensity) [23]. Zou et al. used a dataset of 60,000 randomly selected images from 25 CE videos to create a deep convolutional neural network (DCNN) model and classify digestive organs [24]. However, both of these studies did not prove their efficacy in finding a transitional area because they only analyzed each single frame image and did not prove the effect applied to an actual image. Two deep learning models using pretrained models (VGG16, ResNet 18, and GoogLENet) showed excellent performance [25,26], but both studies also have limitations with respect to their application in real videos using only well-chosen images by expert doctors or using annotated images.

We attempted GI organ classification by finding the transitional area using the characteristic that CE moves in only one direction. Finding the transitional area is time-consuming because images must be reviewed repeatedly if landmarks are not adequately captured or the bowel’s preparation is poor. In a close-up view, gastric intestinal metaplasia could be hard to distinguish from small bowel mucosa. It is difficult to accurately observe the mucosa membrane in a poorly prepared and collapsed bowel. Our AI model’s class activation maps primarily catch mucosa; therefore, the inability to accurately capture mucosa can cause problems in appropriately identifying GI organs (Figure 5).

Consequently, a technique that can process repeatedly captured incorrect images is required for usage in actual CE videos. We extracted images evenly over time to minimize the loss of information and assumed that they could represent the full-length video. We used a noise control method to filter out images with inadequate bowel preparation since high-quality images have a more excellent diagnostic power [27,28].

Even if the accuracy of the AI model reaches 98%, the results for hundreds of thousands of images reveal high heterogeneity, resulting in numerous transitional areas inside one organ (Figure 6a). Son et al. showed excellent performance in distinguishing SB from the entire video by dramatically reducing misclassified images using temporal filtering [29]. On the other hand, we visualized the GI classification results and made it much more practical to find transitional areas, which became more apparent after adjusting the cut-off value of the AI score (Figure 6b, Figure 7 and Figure 8). The cut-off of the AI score can be calibrated according to various circumstances, such as the condition of bowel preparation and GI motility. Although our AI model was trained on the normal CE dataset, it demonstrated excellent performance in CE cases with abnormal lesions such as inflammation, bleeding, abnormal vascular lesions, and polyps (Table 3).

Our study automated the process of extracting image datasets necessary for model development and validation according to specific rules, unlike previous studies in which human experts spent time selecting high-quality images to create datasets [25,26]. A dataset composed of overly refined images has a problem in that it cannot be applied to actual capsule endoscopy videos filled with low-quality frames. We were able to develop AI models with high accuracy without the effort of selecting data, and in the actual CE video application, we were able to overcome poor performance due to inappropriate photos by adjusting AI score cut-offs.

We used the no-code platform, Neuro-T, which is an automated machine learning (ML) tool for model development. The automated ML technique is a novel tool that automatically solves hyperparameter optimization, automated feature engineering, pipeline optimization, and neural architecture search without human experts. Automated ML is also being actively developed by Google and Apple, and its performance has already been proven in over 100 papers in various medical fields [30]. The automated ML algorithm showed high accuracy and performance in classifying dental implant systems that are comparable to dental professionals [19] and improved the professional’s performance [30]. In another study, a model built with automated ML showed the best accuracy compared to other models built using three DCNN architectures (VGGNet-19, GoogLeNet, and Inception-v3) [22]. The performance of the automated ML model (NeuroT) was also the best compared to traditional models in predicting algorithms for the SM invasion of gastric neoplasm [20,31] and the histology of colon polyps based on endoscopic images [21,32].

Our model showed a limitation with respect to poor performance in the esophagus and colon due to data imbalance (Table 3). As CE passes through the esophagus’ narrow lumen, the mucous membrane sticks closely to the camera lens, producing overly close images and frequent misinterpretations with the gastric mucosa or colon. Another reason is that the esophagus is shorter than other GI organs, and the colon contains a substantial amount of feces, which limited model training due to the lack of adequate data. However, these findings may have a minor impact on clinical utilization due to the low relevance of the esophagus and colon observation in small bowel CE, and this can be compensated by adjusting the AI score’s threshold (Table 2).

In this study, we did not provide a computational definition of the GI organ transitional area. As a result, our AI model did not fully automate locating the transitional area. However, we enabled physicians to quickly identify the transitional area by visualizing the outcome of GI organ classification. This method will help reduce the reading time of physicians in practice, and future comparative studies should demonstrate it.

In conclusion, the GI organ classification AI model using a no-code platform demonstrated high accuracy with respect to a CE video. In addition, our method of adjusting the AI score cut-off and visualizing the results provided an intuitive method for locating the gastrointestinal transitional area.

## Figures and Tables

**Table 1:** Summary of the GI organ classification AI model’s performance using a no-code platform.

Organ	Accuracy	Recall	Specificity	Precision	NPV	F1 Score	AUROC	95% CI
Esophagus	0.99	0.97	0.99	0.66	1.0	0.78	0.998	0.996–1.000
Stomach	0.97	0.94	0.99	0.97	0.98	0.96	0.995	0.994–0.996
Small bowel	0.99	1.0	0.99	0.97	1.0	0.98	1.000	1.000–1.000
Colon	0.98	0.96	0.99	0.98	0.97	0.97	0.995	0.995–0.996
Total	0.98	0.97	0.99	0.89	0.99	0.92	0.941	0.935–0.947

GI, Gastrointestinal; AI, artificial intelligence; NPV, negative predictive value; AUROC, area under the receiver operating characteristic curve; CI, confidence interval.

**Table 2:** Video application of the performance of the GI organ classification AI model.

AI Score Threshold	GI Organ	Accuracy	Recall	Specificity	Precision	NPV	F1 Score
0	Esophagus (*n* = 5340)	0.98 (0.97–0.99)	0.96 (0.93–0.98)	0.98 (0.97–0.99)	0.26 (0.19–0.32)	1.0 (1.0–1.0)	0.33 (0.26–0.40)
	Stomach (*n* = 217,974)	0.96 (0.95–0.97)	0.89 (0.86–0.92)	0.98 (0.96–0.99)	0.85 (0.81–0.90)	0.97 (0.96–0.98)	0.85 (0.81–0.88)
	Small bowel (*n* = 987,613)	0.87 (0.85–0.89)	0.83 (0.80–0.85)	0.98 (0.97–0.99)	0.98 (0.97–0.99)	0.68 (0.63–0.72)	0.89 (0.88–0.91)
	Colon (*n* = 193,276)	0.87 (0.85–0.89)	0.91 (0.86–0.96)	0.88 (0.86–0.89)	0.54 (0.48–0.61)	0.97 (0.95–0.99)	0.64 (0.58–0.70)
	Overall (*n* = 1,404,203)	0.92 (0.91–0.93)	0.89 (0.88–0.91)	0.96 (0.95–0.96)	0.67 (0.63–0.71)	0.90 (0.88–0.92)	0.69 (0.65–0.72)
≥99.9%	Esophagus (*n* = 4135)	0.99 (0.98–1.0)	0.98 (0.95–1.0)	0.99 (0.98–1.0)	0.71 (0.63–0.79)	1.0 (1.0–1.0)	0.76 (0.69–0.84)
	Stomach (*n* = 128,737)	0.98 (0.96–1.0)	0.95 (0.92–0.98)	0.98 (0.96–1.0)	0.95 (0.92–0.98)	0.99 (0.99–1.0)	0.94 (0.91–0.97)
	Small bowel (*n* = 638,053)	0.94 (0.93–0.96)	0.92 (0.90–0.94)	0.99 (0.99–1.0)	1.0 (0.99–1.0)	0.83 (0.80–0.87)	0.95 (0.94–0.97)
	Colon (*n* = 133,981)	0.94 (0.92–0.96)	0.94 (0.89–0.99)	0.94 (0.93–0.96)	0.73 (0.66–0.79)	0.99 (0.97–1.0)	0.82 (0.77–0.87)
	Overall (*n* = 904,906)	0.96 (0.96–0.97)	0.94 (0.93–0.96)	0.98 (0.97–0.99)	0.85 (0.82–0.88)	0.95 (0.94–0.96)	0.87 (0.85–0.90)

GI, gastrointestinal; AI, artificial intelligence; NPV, negative predictive value; AI score, predictive probability; Values with 95% confidence intervals are described in parentheses.

**Table 3:** Performance of GI organ classification in AI model by small bowel diseases.

AI Score Threshold	Diseases	Accuracy	Recall	Specificity	Precision	NPV	F1 Score
0	Normal (*n* = 390,410)	0.93 (0.91–0.95)	0.90 (0.88–0.93)	0.96 (0.94–0.97)	0.67 (0.60–0.74)	0.91 (0.87–0.94)	0.70 (0.64–0.75)
	Blood (*n* = 336,656)	0.89 (0.86–0.91)	0.83 (0.77–0.88)	0.93 (0.91–0.95)	0.58 (0.48–0.67)	0.89 (0.84–0.93)	0.57 (0.48–0.66)
	Inflamed (*n* = 250,158)	0.93 (0.91–0.95)	0.91 (0.89–0.94)	0.97 (0.95–0.98)	0.72 (0.64–0.80)	0.90 (0.85–0.94)	0.73 (0.66–0.80)
	Vascular (*n* = 239,632)	0.93 (0.91–0.95)	0.90 (0.86–0.93)	0.96 (0.94–0.98)	0.69 (0.59–0.78)	0.89 (0.83–0.94)	0.70 (0.62–0.78)
	Polypoid (*n* = 187,347)	0.94 (0.92–0.96)	0.94 (0.92–0.97)	0.96 (0.95–0.98)	0.72 (0.62–0.81)	0.92 (0.88–0.96)	0.74 (0.66–0.82)
≥99.9%	Normal (*n* = 261,234)	0.96 (0.95–0.98)	0.96 (0.94–0.98)	0.97 (0.96–0.99)	0.85 (0.81–0.90)	0.96 (0.94–0.98)	0.88 (0.85–0.92)
	Blood (*n* = 196,127)	0.95 (0.92–0.97)	0.88 (0.83–0.94)	0.97 (0.95–0.99)	0.74 (0.65–0.83)	0.94 (0.92–0.97)	0.76 (0.68–0.85)
	Inflamed (*n* = 168,331)	0.97 (0.96–0.98)	0.96 (0.94–0.98)	0.98 (0.97–1.0)	0.92 (0.88–0.96)	0.94 (0.90–0.97)	0.93 (0.89–0.96)
	Vascular (*n* = 153,850)	0.98 (0.97–0.99)	0.96 (0.93–0.98)	0.99 (0.98–1.0)	0.86 (0.78–0.93)	0.95 (0.91–0.98)	0.88 (0.83–0.94)
	Polypoid (*n* = 125,364)	0.97 (0.96–0.99)	0.98 (0.96–1.0)	0.98 (0.97–1.0)	0.91 (0.86–0.96)	0.97 (0.94–0.99)	0.93 (0.89–0.97)

GI, Gastrointestinal; AI, artificial intelligence; NPV, negative predictive value; AI score, predictive probability; Values with 95% confidence intervals are described in parentheses.

## Data Availability

Not applicable.

## Supplementary Materials

The following supporting information can be downloaded at: https://www.mdpi.com/article/10.3390/diagnostics13081389/s1. Table S1. Number of images extracted from 100 CE videos and used for validating GI organ classification AI model after the noise control process; Table S2. Comparisons of performance before and after 99.9% cut-off application by paired T-test; Table S3. Comparisons of GI organ-specific accuracy between small bowel diseases by independent T-test.
