# Supplementary material for: Automatic Classification of GI Organs in Wireless Capsule Endoscopy Using a No-Code Platform-Based Deep Learning Model
Source: Diagnostics (Basel). 2023 Apr 11;13(8):1389. doi: 10.3390/diagnostics13081389 (PMC10137357; doi:10.3390/diagnostics13081389)
Supplement: Supplementary file 1 [file diagnostics-13-01389-s001.zip › diagnostics-2268484-supplementary.pdf]

Supplementary Table S1. Number of images extracted from 100 CE videos and used for validating GI organ classification AI model after the noise control process

	Total GI tracts	Esophagus	Stomach	Small bowel	Colon
Extracted images	1,715,852	5,400	229,618	1,206,033	274,801
(Range of images/case)	(5,597-50,720)	(0-2,752)	(0-14,367)	(1,583-32,617)	(0-22,040)
Noise images	311,525 (18.2%)	60 (11.1%)	11,644 (5.07%)	218,420 (18.1%)	81,525 (29.67%)
(Range of images/case)	(51-35,181)	(0-23)	(0-2,378)	(9-19,195)	(0-15,142)
Total clear images	1,404,203	5,340	217,974	987,613	193,276
(Range of images/case)	(5,546-27,437)	(0-2,729)	(0-12,631)	(1,243-24,197)	(0-12,490)

Supplementary Table S2. Comparisons of performance before and after 99.9% cut-off application by paired T-test

t-score		Accuracy	Recall	Specificity	Precision	NPV	F1 score
99.9%/baseline	Esophagus	**4.017 (p<0.001)**	1.220 (p=0.226)	**3.952 (p<0.001)**	**11.851 (p<0.001)**	0.867 (p=0.388)	**12.689 (p<0.001)**
	Stomach	**2.136 (p=0.035)**	**7.769 (p<0.001)**	0.568 (p=0.572)	**5.490 (p<0.001)**	**4.935 (p<0.001)**	**6.848 (p<0.001)**
	Small bowel	**16.712 (p<0.001)**	**18.893 (p<0.001)**	**4.045 (p<0.001)**	**3.007 (p=0.003)**	**13.516 (p<0.001)**	**15.958 (p<0.001)**
	Colon	**13.495 (p<0.001)**	**3.216 (p=0.002)**	**16.248 (p<0.001)**	**11.064 (p<0.001)**	**3.077 (p=0.003)**	**11.332 (p<0.001)**
	Total	**12.853 (p<0.001)**	**11.309 (p<0.001)**	**7.036 (p<0.001)**	**13.008 (p<0.001)**	**10.597 (p<0.001)**	**14.842 (p<0.001)**

Supplementary Table S3. Comparisons of GI organ-specific accuracy between small bowel diseases by independent T-test

t-score	Normal					Blood		Inflamed		Vascular
	**Blood**	**Inflamed**	**Vascular**	**Polypoid**	**Inflamed**	**Vascular**	**Polypoid**	**Vascular**	**Polypoid**	**Polypoid**
**Baseline**										
**Esophagus**	**2.470**	-0.632	0.302	**-2.032**	**-2.549**	**-2.409**	**-2.665**	0.781	-0.918	-1.618
	**(p=0.024)**	(p=0.531)	(p=0.764)	**(p=0.049)**	**(p=0.02)**	**(p=0.026)**	**(p=0.016)**	(p=0.441)	(p=0.366)	(p=0.126)
**Stomach**	2.630	0.505	0.124	**-1.041**	**-2.298**	**-2.379**	**-3.193**	-0.282	-1.451	1.037
	(p=0.015)	(p=0.616)	(p=0.902)	**(p=0.304)**	**(p=0.03)**	**(p=0.025)**	**(p=0.005)**	(p=0.78)	(p=0.156)	(p=0.309)
**Small bowel**	0.418	0.322	-0.179	-0.715	-0.046	-0.517	-1.011	-0.401	-0.821	-0.471
	(p=0.678)	(p=0.749)	(p=0.859)	(p=0.478)	(p=0.963)	(p=0.609)	(p=0.319)	(p=0.691)	(p=0.418)	(p=0.641)
**Colon**	0.837	-0.606	-0.177	-0.564	-1.198	-0.906	-1.151	0.371	0.029	-0.322
	(p=0.411)	(p=0.548)	(p=0.861)	(p=0.576)	(p=0.242)	(p=0.374)	(p=0.26)	(p=0.714)	(p=0.977)	(p=0.75)
**Overall**	**2.735**	-0.067	-0.172	-0.798	**-2.529**	**-2.603**	**-3.147**	-0.096	-0.648	-0.549
	**(p=0.007)**	(p=0.947)	(p=0.864)	(p=0.426)	**(p=0.013)**	**(p=0.010)**	**(p=0.002)**	(p=0.923)	(p=0.518)	(0.584)
**AI score ≥ 99.9**										
**Esophagus**	1.675	-1.015	-0.269	-1.009	-1.705	-1.684	-1.460	0.694	-0.128	-0.741
	(p=0.111)	(p=0.316)	(p=0.789)	(p=0.319)	(p=0.105)	(p=0.109)	(p=0.154)	(p=0.493)	(p=0.899)	(p=0.465)
**Stomach**	0.278	-0.784	-0.664	-0.800	-1.357	-1.178	-1.545	-0.038	-0.778	-0.887
	(p=0.782)	(p=0.437)	(p=0.511)	(p=0.428)	(p=0.191)	(p=0.248)	(p=0.14)	(p=0.97)	(p=0.442)	(p=0.383)
**Small bowel**	-0.272	0.869	-0.352	-0.245	1.046	-0.145	-0.058	-0.967	-0.789	0.039
	(p=0.786)	(p=0.389)	(p=0.727)	(p=0.808)	(p=0.302)	(p=0.886)	(p=0.954)	(p=0.341)	(p=0.435)	(p=0.97)
**Colon**	0.861	-0.695	-0.695	-0.315	-1.213	-1.019	-0.918	-0.077	0.219	0.244
	(p=0.399)	(p=0.491)	(p=0.492)	(p=0.754)	(p=0.24)	(p=0.318)	(p=0.366)	(p=0.939)	(p=0.828)	(p=0.809)
**Overall**	1.251	-0.491	-1.000	-0.821	-1.600	**-2.260**	-1.899	-0.738	-0.455	0.176
	(p=0.212)	(p=0.624)	(p=0.319)	(p=0.413)	(p=0.099)	**(p=0.026)**	(p=0.060)	(p=0.462)	(p=0.650)	(p=0.860)
